# Learning to walk with an adaptive gain proportional myoelectric controller for a robotic ankle exoskeleton

**DOI:** 10.1186/s12984-015-0086-5

**Published:** 2015-11-04

**Authors:** Jeffrey R. Koller, Daniel A. Jacobs, Daniel P. Ferris, C. David Remy

**Affiliations:** Department of Mechanical Engineering, University of Michigan, 2350 Hayward, Ann Arbor, 48109 MI USA; School of Kinesiology, University of Michigan, 1402 Washington Heights, Ann Arbor, 48109 MI USA

**Keywords:** Exoskeleton, Adaptive, Ankle, Metabolic, Rehabilitation, Gait

## Abstract

**Background:**

Robotic ankle exoskeletons can provide assistance to users and reduce metabolic power during walking. Our research group has investigated the use of proportional myoelectric control for controlling robotic ankle exoskeletons. Previously, these controllers have relied on a constant gain to map user’s muscle activity to actuation control signals. A constant gain may act as a constraint on the user, so we designed a controller that dynamically adapts the gain to the user’s myoelectric amplitude. We hypothesized that an adaptive gain proportional myoelectric controller would reduce metabolic energy expenditure compared to walking with the ankle exoskeleton unpowered because users could choose their preferred control gain.

**Methods:**

We tested eight healthy subjects walking with the adaptive gain proportional myoelectric controller with bilateral ankle exoskeletons. The adaptive gain was updated each stride such that on average the user’s peak muscle activity was mapped to maximal power output of the exoskeleton. All subjects participated in three identical training sessions where they walked on a treadmill for 50 minutes (30 minutes of which the exoskeleton was powered) at 1.2 ms^-1^. We calculated and analyzed metabolic energy consumption, muscle recruitment, inverse kinematics, inverse dynamics, and exoskeleton mechanics.

**Results:**

Using our controller, subjects achieved a metabolic reduction similar to that seen in previous work in about a third of the training time. The resulting controller gain was lower than that seen in previous work (*β*=1.50±0.14 versus a constant *β*=2). The adapted gain allowed users more total ankle joint power than that of unassisted walking, increasing ankle power in exchange for a decrease in hip power.

**Conclusions:**

Our findings indicate that humans prefer to walk with greater ankle mechanical power output than their unassisted gait when provided with an ankle exoskeleton using an adaptive controller. This suggests that robotic assistance from an exoskeleton can allow humans to adopt gait patterns different from their normal choices for locomotion. In our specific experiment, subjects increased ankle power and decreased hip power to walk with a reduction in metabolic cost. Future exoskeleton devices that rely on proportional myolectric control are likely to demonstrate improved performance by including an adaptive gain.

**Electronic supplementary material:**

The online version of this article (doi:10.1186/s12984-015-0086-5) contains supplementary material, which is available to authorized users.

## Background

In order to achieve optimal assistance, the controller of an active prosthetic or orthotic device must accomplish three tasks. It must reliably determine the user’s *intent*, precisely coordinate the *timing* of assistance with the user, and provide actuation profiles of a suitable *shape*. Only if the controller succeeds in all three tasks, the robotic device can achieve its assistive goal. For example, many robotic assistive devices aim to minimize the energetic cost for the user to perform a given task. Any amount of error in the controller’s intent recognition, timing, or actuation shape can result in motion that is energetically costly, unnatural, or potentially dangerous for the user [1].

Without direct access to the human nervous system, many lower-limb assistive robotic devices detect intent and timing from estimates of the user’s motion. These measurements are called *mechanically intrinsic* as they are taken from the mechanical device itself. These measurements are used to estimate intent and trigger the timing of predefined actuation profiles whose shapes correspond to estimates of intended motion [2]. Controllers that rely on mechanically intrinsic measurements often use joint angles, impedances, gait events, or force measurements from the device to control actuation [3–7]. Recent exoskeleton controller designs relying on this type of sensing have shown promise in reducing the user’s metabolic cost during walking [8, 9]. However, using mechanically intrinsic measurements for control has fundamental limitations. Because mechanically intrinsic measurements are outcomes of physical motion, they are prone to mechanical delays. The desired movement has already started by the time the controller senses it. This delay can cause the control timing to lag behind the user and result in the user fighting the device [10]. Furthermore, the measurements are subject to complex interactions between the user’s musculoskeletal system and the mechanical structure of the device. If the combined human-machine dynamics are not well understood it can be difficult to reliably estimate intent. Additionally, it is impossible for the user to receive appropriate assistance for motion outside of the controller’s intent laws since all actuation profile shapes are predefined for specific movements.

The drawbacks of relying on mechanically intrinsic measurements can potentially be overcome by a direct access to the user’s nervous system. One approach using bioelectrical signals for control is proportional myoelectric control. A proportional myoelectric controller sends a control signal to the actuators that is proportional to the muscle recruitment of the user [11, 12]. In these controllers muscle recruitment is measured using electromyography (EMG). The controller makes no assumptions about the human-machine dynamics because the measurements used to determine intent come straight from the user instead of the device. This puts the user in direct control of the exoskeleton and allows for intent recognition to be accurate and consistent. Additionally, proportional myoelectric control has the potential for zero lag in timing behind the user due to the electromechanical delay of EMG [13]. EMG signals are produced before muscle tension develops which allows a proportional myoelectric controller to have a buffer of time between sensor measurement and actuation. The control signal shape of these controllers is proportional to the user’s EMG signal meaning there is inherent human-machine synchronization. Additionally, this proportionality implies that the device is not limited to predefined actuation profiles. Our research group has shown that proportional myoelectric control is a viable control method for lower-limb robotic exoskeletons that produces a relatively natural and economical gait [14–19].

**Fig. 1:**
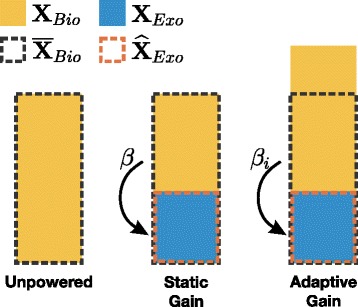
Representation of Proportional Myoelectric Control. The above figure is a graphical representation to compliment the mathematical theory that describes proportional myoelectric control. In all of the bar graphs, *X*_*Tot*_ is represented by the summation of *X*_*Bio*_ and *X*_*Exo*_

A proportional myoelectric control scheme can be illustrated as follows and is graphically represented by Fig. 1. Suppose **X**_*Tot*_ represents the total actuator activation at the assisted joint including both biological muscles and the exoskeleton’s mechanical actuators. When walking in an exoskeleton, we can apportion the activity from the biological joint as **X**_*Bio*_ and the activity from the exoskeleton as **X**_*Exo*_. The biological activity can be thought of as muscle activity about the assisted joint measured via EMG and the exoskeleton activity can be thought of as the control signals being sent to the exoskeleton actuators.

(1) $$\begin{array}{*{20}l} \mathbf{X}_{Tot} = \mathbf{X}_{Bio} + \mathbf{X}_{Exo}  \end{array} $$

In a proportional myoelectric controller, the activity from the exoskeleton is proportional to the biological activity by some gain *β*. *β* maps biological activity to exoskeleton activity. This mapping is scaled by the ratio $c = {\mathbf {\widehat {{X}}}_{\textit {Exo}}}/{\mathbf {\overline {X}}_{\textit {Bio}}}$, where $\mathbf {\widehat {X}}_{\textit {Exo}}$ is the maximum unsaturated exoskeleton activity and $\mathbf {\overline {X}}_{\textit {Bio}}$ is the normal unassisted joint activity.

(2) $$\begin{array}{*{20}l} \mathbf{X}_{Exo} = \beta \left(\frac{\mathbf{\widehat{{X}}}_{Exo}}{\mathbf{\overline{X}}_{Bio}} \right)\mathbf{X}_{Bio} = \beta \cdot c \cdot \mathbf{X}_{Bio}  \end{array} $$

The exoskeletons presented here and in our previous research can provide about half the power of the normal unassisted joint, so *c* has been estimated as *c*≈0.5 [14, 15].

In the past, the proportional myoelectric controllers developed by our group created a control signal for actuation by using a *constant* gain of *β*=2 to map the EMG linear envelope to an actuation voltage. This gain was chosen with the assumption that during powered walking total joint activity should be equal to the unassisted joint activity: $\mathbf {X}_{\textit {Tot}} = \mathbf {\overline {X}}_{\textit {Bio}}$. Additionally, this gain was meant to allow maximal assistance ($\mathbf {X}_{\textit {Exo}} = \mathbf {\widehat {X}}_{\textit {Exo}}$) during steady state operation. With this, we got from Eq. 1:

(3) $$\begin{array}{*{20}l} \mathbf{\overline{X}}_{Bio} = \mathbf{X}_{Bio} + \mathbf{\widehat{X}}_{Exo} = \mathbf{X}_{Bio} + \mathbf{\overline{X}}_{Bio} \cdot c,  \end{array} $$

and thus a reduction in biological joint activity: $\mathbf {X}_{\textit {Bio}} = \left (1-c \right) \mathbf {\overline {X}}_{\textit {Bio}}$. For $\mathbf {X}_{\textit {Exo}} = \mathbf {\widehat {X}}_{\textit {Exo}}$, we can solve Eq. 2 for the necessary *β*:

(4) $$\begin{array}{*{20}l} \beta =\frac{\mathbf{\overline{X}}_{Bio}}{\mathbf{X}_{Bio}} = \frac{1}{1-c} = \frac{1}{1-0.5}= 2.  \end{array} $$

In previous work, this choice of *β* resulted in large reductions in metabolic cost. Our studies have also shown that subjects indeed attempted to adapt to $\mathbf {X}_{\textit {Bio}}\approx \frac {1}{\beta }\mathbf {\overline {X}}_{\textit {Bio}}$, in accordance with Eq. 4 [15].

Yet walking in an exoskeleton is different than unassisted walking and we might prefer more or less total joint activity than $\mathbf {\overline {X}}_{\textit {Bio}}$. In these previous studies, subjects had the ability to adapt $\mathbf {X}_{\textit {Bio}}<\frac {1}{\beta }\mathbf {\overline {X}}_{\textit {Bio}}$ and deliberately chose not to. This result suggests that $\mathbf {X}_{\textit {Tot}}<\mathbf {\overline {X}}_{\textit {Bio}}$ is not energetically economical since we generally adapt to move with as little energy as possible [20–24]. However, subjects were somehow constrained when attempting $\mathbf {X}_{\textit {Bio}}>\frac {1}{\beta }\mathbf {\overline {X}}_{\textit {Bio}}$ as they would saturate the exoskeleton. Previous work has shown that subjects avoided this saturation limit, but we do not know the exact reason why. Perhaps subjects avoided saturation due to discomfort or possibly they chose to avoid the increased cognitive complexity that comes with learning a highly nonlinear task. Whatever the reason, we know that subjects naturally chose to avoid operating the exoskeleton within the saturation range. From Eq. 2 it follows that

(5) $$\begin{array}{*{20}l} \mathbf{X}_{Exo} & = \beta \left(\frac{\mathbf{\widehat{{X}}}_{Exo}}{\mathbf{\overline{X}}_{Bio}} \right)\mathbf{X}_{Bio} > \mathbf{\widehat{X}}_{Exo}\text{, for }\mathbf{X}_{Bio}>\frac{1}{\beta}\mathbf{\overline{X}}_{Bio},  \end{array} $$

so it is unclear whether $\mathbf {X}_{\textit {Tot}}=\mathbf {\overline {X}}_{\textit {Bio}}$ is truly the optimal value, or if subjects would prefer a larger **X**_*Tot*_ if saturation were avoidable.

Therefore, we saw the need for a proportional myoelectric controller that allows users to explore higher magnitudes of total joint activity. Such a controller would allow users to adapt to find the most energetically economical gait on their own. This adapation could potentially answer whether or not $\mathbf {\overline {X}}_{\textit {Bio}}$ is the energetically optimal total joint activity for walking in an exoskeleton. In designing such a proportional myoelectric controller, we wanted to keep the exoskeleton performing at maximum potential regardless of biological activity (i.e., $\mathbf {X}_{\textit {Exo}}=\mathbf {\widehat {X}}_{\textit {Exo}}$). This design would allow for the user to vary the total joint activity by just varying their biological activity. We made this possible by designing a proportional myoelectric controller in which the gain was free to *dynamically* adapt on a stride by stride basis. In other words, *β* was no longer held constant but adapted itself on each stride *i* to maintain maximal exoskeleton output. If we set $\mathbf {X}_{\textit {Exo}}=\mathbf {\widehat {X}}_{\textit {Exo}}$ in Eq. 2, we can express *β*_*i*_ as follows:

(6) $$\begin{array}{*{20}l} \beta_{i} = \frac{\mathbf{\overline{X}}_{Bio}}{\mathbf{X}_{Bio,i}}.  \end{array} $$

Combining Eqs. 1, 2 and 6 shows that this adaptive proportional myoelectric controller could allow users to vary their amount of total joint activity:

(7) $$\begin{array}{*{20}l} \mathbf{X}_{Tot} = \frac{\mathbf{\overline{X}}_{Bio}}{\beta_{i}}+\mathbf{\widehat{X}}_{Exo}.  \end{array} $$

It is notable that lower gains *β*_*i*_ (a consequence of larger **X**_*Bio*,*i*_) result in larger values for **X**_*Tot*_. A time series representation of this controller dynamically adapting to the user is shown in Additional file 1: Figure A1.

The purpose of this study was to to test the performance of an adaptive proportional myoelectric controller on a robotic ankle exoskeleton. This controller allowed users to explore a greater possible parameter space of walking in an exoskeleton compared to walking with traditional proportional myoelectric controllers. We were interested in what *β* gain user’s choose when provided an adaptive controller. We tested young healthy subjects walking with the adaptive gain proportional myoelectric controller on bilateral robotic ankle exoskeletons. We predicted that the adaptive controller would allow users to walk with reduced energetic cost and a *β* gain less than that of our previous work with a constant gain controller. A *β* gain less than that of our previous work would indicate that subjects have adapted to using more total ankle activity than that of unassisted walking.

## Methods

### Subjects

We tested eight healthy subjects for this study (male, 21 ± 1 years, 74.0 ± 2.7 kg, 180.0 ± 2.8 cm; means ± s.e.m.). All subjects were prescreened for exoskeleton hardware fit prior to testing. Subjects exhibited no gait abnormalities and had no prior experience walking in a powered exoskeleton. Prior to testing, all subjects gave informed written consent to participate in the study in accordance to the University of Michigan’s Medical School’s Institutional Review Board (HUM00070022).

### Exoskeleton hardware

We custom fabricated bilateral ankle exoskeletons for this study similar to those used in previous studies from our research group [14, 15, 25, 26]. The exoskeletons consisted of a shank component and a shoe component that were joined by a rotational joint. This joint constrained the exoskeleton motion to plantar flexion and dorsiflexion. The shank was made from stainless steel rods and plastic cuffs. The shoe was a standard orthotic shoe that was outfitted with attachments for actuation. The exoskeleton could accommodate subjects that wore between a 9 and 11 U.S. men’s shoe size.

We actuated the exoskeletons using custom built artificial pneumatic muscles attached posteriorly allowing for plantar flexion assistance when actuated [25]. We attached a load cell in series (Omega Engineering, Stamford, Connecticut) with the actuator to record actuation kinetics. The shoe, shank, actuator, and load cell combined to a total mass of 2.08 kg (approximately 0.81 kg at the foot and 1.27 kg at the shank).

### Exoskeleton control

The exoskeleton controller was a dynamically adaptive proportional myoelectric controller. We used the wearer’s soleus EMG for the input signal to the controller in order to maintain biological synergy with the exoskeleton.

**Fig. 2:**
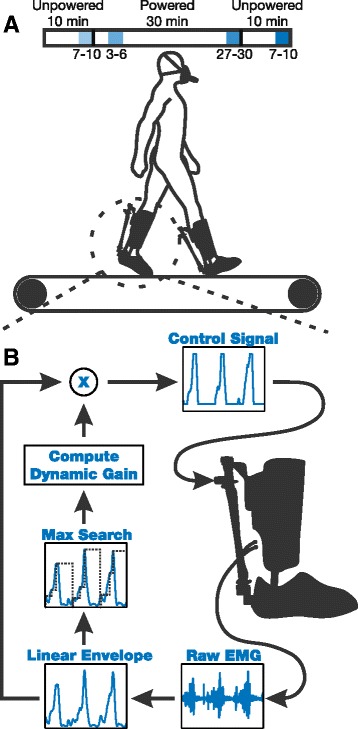
Testing Protocol and Control Scheme. **a** All eight subjects walked at 1.2 ms^-1^ with the exoskeletons on during three separate training sessions. Each session consisted of 50 minutes of walking where the first 10 minutes were unpowered, the following 30 minutes were powered, and the last 10 minutes were unpowered. Four time intervals from each session were analyzed in the analysis: minutes 7–10 of the 1^st^ unpowered section, minutes 3–6 of the powered section, minutes 27–30 of the powered session, and minutes 7-10 of the 2^nd^ unpowered section. **b** For control we processed the soleus linear envelope in real time and then conducted a maximum search on a stride by stride basis. We used these max values to calculate the mapping gain that the linear envelope was multiplied by to create the actuation control signal

We designed the controller to process the user’s raw soelus EMG into its linear envelope in real time. The processing consisted of a high-pass filter (2nd order Butterworth, cutoff frequency 80 Hz) to remove motion artifacts, followed by full wave rectification. We then low-pass filtered the rectified signal (2nd order Butterworth, cutoff frequency 4 Hz) to get the linear envelope. In a traditional proportional myoelectric controller, this linear envelope would then be multiplied by a static mapping gain to calculate the control signal [11, 12]. In the current study, this mapping gain was dynamically adjusted by the controller using the following methodology (Fig. 2b).

For each stride *i*, we determined the maximum voltage of the linear envelope, *x*_*i*_, in real time. We then calculated the gain, *g*_*i*_, necessary for this value to reach a desired peak actuation voltage, *V*_*peak*_.

(8) $$\begin{array}{*{20}l} g_{i} = \frac{V_{peak}}{x_{i}}  \end{array} $$

We calculated the dynamic gain, *G*_*i*_, using a finite impulse response (FIR) filter with a tap size, *N*=50, and unity weighting. *G*_*i*_ was then used to proportionally scale the EMG linear envelope to the actuation control signals.

(9) $$\begin{array}{*{20}l} G_{i} = \frac{1}{N} \left(\sum\limits_{j = i-N}^{i-1}g_{j} \right)  \end{array} $$

The actuation control signals were sent to proportional pressure control valves (MAC Valves, Wixom, MI). These valves regulated the pressure in the artificial pneumatic muscles to be proportional to the user’s amplified linear envelope. This pressure roughly corresponded to the exoskeleton torque output with some nonlinearities induced by actuator dynamics and a changing moment arm. We ran our controller on a desktop and real-time control board (dSPACE, Inc., Northville, MI) during all testing. All software was composed in Simulink (The Mathworks, Inc., Natick, MA) and then converted to ControlDesk (dSPACE, Inc., Northville, MI) using commercial dSPACE software.

### Testing protocol

The following protocol is largely adapted from [15]. All subjects participated in three identical training sessions with the device (sessions 1–3). We conducted these training sessions over the course of 7–14 days for each subject, allowing at least one day rest between sessions for motor consolidation [14, 27]. Each training session consisted of 50 continuous minutes of level ground walking in the exoskeleton. Subjects walked on a split belt treadmill at 1.2 ms^-1^ (Bertec Corporation, Columbus, OH) for all tests. The first 10 minutes of each walking session were with the device unpowered (i.e., no actuation). We gave subjects a verbal warning before actuation was turned on for the following 30 minutes. The FIR filter was initialized with zeros, so a peak control signal was not reached until 50 strides or approximately 60 seconds of powered walking. After the full 30 minutes of powered walking, we gave subjects a verbal warning before actuation was turned off for another 10 minutes.

We analyzed data from four time windows of each session: minutes 7–10 of the 1^st^ unpowered condition, minutes 3–6 of the powered condition, minutes 27–30 of the powered condition, and minutes 7-10 of the 2^nd^ unpowered condition (Fig. 2a). Respiratory data was averaged over each three minute time window. Gait data was averaged over the last 25 strides of each time window. From this gait data, we calculated muscle recruitment, inverse kinematics, inverse dynamics, and exoskeleton mechanics. Strides were defined as heel-strike (0 % gait cycle) to heel-strike (100 % gait cycle). Data from all session’s 1^st^ unpowered condition were averaged to get the *Average Unpowered* values. These values are compared to data from the end of powered conditions of each session in Figs. 4 through 8.

### Metabolic cost

We used a portable open-circuit indirect spirometry system (CareFusion Oxycon Mobile, Hoechberg, Germany) to measure *O*_2_ and *CO*_2_ flow rates. We used formulas from Brockway [28] to convert these measurements to metabolic power. Prior to walking trials, we recorded a three minute standing trial from each subject. We averaged over these three minutes to get each subject’s standing metabolic power which was then subtracted from each walking trial to calculate the net metabolic power of each walking condition [29]. We analyzed each walking condition by averaging the metabolic power over a three minute interval then normalizing it by the subjects body mass. During testing, we monitored each subject’s respiratory exchange ratio (RER) to ensure that it remained in the aerobic range (RER <1) [30].

### Electromyography

We measured electromyography from the soleus, tibialis anterior, medial gastrocnemius, biceps femoris long head, vastus lateralis, and rectus femoris. All EMG recordings came from the subject’s right side except for the soleus in which recordings came from both the left and right since they were used as control inputs. We used bipolar surface electrodes (sample rate: 1000 Hz; Biometrics, Ladysmith, VA) with an inter-electrode distance of 2.0 cm and electrode diameter of 1.0 cm to record all muscle activity. The EMG amplifier used for data collection had a bandwidth of 20–460 Hz. We placed all electrodes according to the procedure of Winter and Yack [31].

For post-processing the EMG data, we high-pass filtered all EMG signals with a 35 Hz cut-off frequency (3rd order Butterworth filter, zero-lag) and then full-wave rectified. We then low-pass filtered all rectified signals with a 10 Hz cut-off frequency (3rd order Butterworth filter, zero lag) to achieve the signal’s linear envelope. Each linear envelope was then epoched by stride (heel-strike to heel-strike) and averaged. We normalized each muscle’s linear envelope by its corresponding peak voltage from the end of the 1^st^ unpowered walking portion of the session [31]. We additionally calculated the root mean square (r.m.s.) stride average for the rectified EMG signal. The r.m.s. calculations were normalized by the average r.m.s. from the end of the 1^st^ unpowered portion of each session. All EMG normalization was done prior to averaging.

### Kinematics

We measured joint kinematics during treadmill walking using a 10-camera motion capture system (sample rate: 100 Hz; Vicon, Oxford, UK). We used a 39 reflective marker set for each subject (34 on the pelvis and lower limbs, 4 on the torso, and 1 on the head). All joint kinematics were calculated from raw marker data using OpenSim 3.2 [32]. In OpenSim we scaled a generic model to subject specific marker placements. The model consisted of lower extremities and a trunk with 23 degrees of freedom and 54 actuators. We ensured that all subject model scaling and inverse kinematic r.m.s. values were within the range recommend by OpenSim during processing [33].

We calculated the Pearson product moment correlations between the mean joint kinematics from the end of powered conditions to the end of 1^st^ unpowered conditions. We assessed similarities in powered verses unpowered joint kinematics by the coefficient of determination (*R*^2^) of these correlations [14].

### Joint mechanics

We imported all ground reaction force data into OpenSim 3.2 to use in conjunction with the calculated joint kinematics to perform inverse dynamics. We scaled each model’s mass anthropomorphically using the subject’s mass and then manually included additional mass at the shank and foot to account for the exoskeleton. We used OpenSim’s residual reduction algorithm (RRA) to iteratively adjust the model as needed to get residual forces and moments as low as possible. We used the adjusted model to calculate inverse dynamics. Our final residuals after using the RRA can be seen in Table 1. These residuals are within OpenSim’s recommended ranges with the exception of *F*_*y*_ maximum and *F*_*z*_ root mean square which are marginally outside of the recommended ranges [33]. We believe these values are acceptable and we attribute the larger residuals to the added complexity of the exoskeleton being present in the analysis.

**Table 1:** Average residual values after final run of the RRA in OpenSim

	*F*_*x*_	*F*_*y*_	*F*_*z*_	*M*_*x*_	*M*_*y*_	*M*_*z*_	pErr _*x*_	pErr _*y*_	pErr _*z*_
	(N)	(N)	(N)	(Nm)	(Nm)	(Nm)	(cm)	(cm)	(cm)
Maximum	12.9	33.3	17.4	29.3	40.6	40.6	3.8	2.3	0.4
Root mean square	7.4	9.6	11.1	9.8	19.3	11.1	2.6	1.5	0.2

*F*_*x*_, *F*_*y*_, and *F*_*z*_ refer to the residual forces at the pelvis. *M*_*x*_, *M*_*y*_, and *M*_*z*_ refer to the residual moments at the pelvis. pErr _*x*_, pErr _*y*_, and pErr _*z*_ refer to the translational position error of the markers

To calculate all joint powers, we multiplied joint angular velocities by the joint torque. We took a simple derivative of the joint positions to get the joint angular velocities and filtered them with a 25 Hz cut-off frequency (3rd order Butterworth, zero-lag) to remove the amplified noise that resulted from taking the derivative. We calculated biological ankle power by subtracting the exoskeleton power from the total ankle power at each time instance. We calculated average joint power values by taking the time interval of the power time series data and dividing it by corresponding stride periods [34, 35]. Average positive and negative power values were computed by separating out the time integrals to periods of positive and negative power. Average net power was calculated using the time series of all power data. Following methodology from [34], we assessed total average positive power, $\overline {P}_{\textit {Tot}}^{+}$, as the sum of average positive power from the ankle, knee, and hip ($\overline {P}_{\textit {Ankle}}^{+}$, $\overline {P}_{\textit {Knee}}^{+}$, $\overline {P}_{\textit {Hip}}^{+}$, respectively).

(10) $$\begin{array}{*{20}l} \overline{P}_{Tot}^{+} = \overline{P}_{Ankle}^{+} + \overline{P}_{Knee}^{+} + \overline{P}_{Hip}^{+}  \end{array} $$

### Exoskeleton mechanics

The distance from the base of the actuator attachment to the exoskeleton joint center was 10.07 cm. Knowing the ankle joint angle from the inverse kinematics, we calculated the moment arm on the actuator at each time instance of collection. We filtered all load cell data with a 25 Hz cut-off frequency (3rd order Butterworth filter, zero-lag). We multiplied the filtered force data by the calculated moment arm to get the exoskeleton torques. The calculated exoskeleton torques were multiplied by the ankle angular velocities to calculate the exoskeleton power. We calculated average exoskeleton power values the same way as average joint power values. We calculated exoskeleton mechanics from one exoskeleton per subject.

### Statistical analyses

We performed two types of repeated-measures ANOVA analysis using SPSS Statistics 22 (IBM, Armock, NY) on all data of interest with a significance level set to 0.05. One ANOVA analysis compared across the four time windows of each training session. Another ANOVA analysis compared across the training sessions of each time window.

## Results

### Metabolic cost

**Fig. 3:**
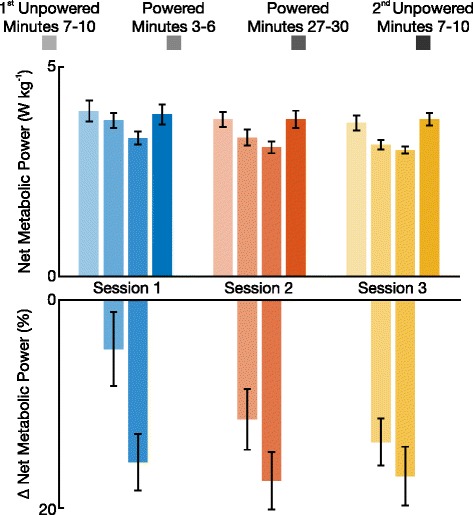
Metabolic Power Reductions. The top axis shows the mean net metabolic power required by eight subjects to walk in the exoskeleton across the three training sessions. All net metabolic power values are normalized by subject mass. The bottom axis represents the powered conditions of this same data as a mean percent change in net metabolic power. Error bars represent ± 1 s.e.m

As subjects began to adapt to the exoskeleton, the amount of metabolic power required to walk in the device decreased (Fig. 3). Subjects had a significant decrease in metabolic power in every session (all *P* <0.05). By the end of powered walking in session 3, subjects were able to walk with a net metabolic power of 3.01±0.08 W kg ^−1^ (mean ± s.e.m., here and throughout). Compared to the first unpowered condition of that same session, 3.66 ± 0.18 W kg ^−1^, this was a reduction of 17.8 %. All net metabolic power values are listed in Table 2.

**Table 2:** Resulting net metabolic cost from each time interval across sessions

	1^st^ Unpowered	Powered	Powered	2^nd^ Unpowered	Within session
	minutes 7–10	minutes 3–6	minutes 27–30	minutes 7–10	*P*-Value
					
Session 1	3.94 ± 0.25	3.72 ± 0.18	3.30 ± 0.15	3.86 ± 0.24	0.002
Session 2	3.75 ± 0.18	3.31 ± 0.19	3.08 ± 0.14	3.75 ± 0.20	0.024
Session 3	3.66 ± 0.18	3.14 ± 0.11	3.01 ± 0.08	3.74 ± 0.15	0.006
Across session *P*-Value	0.070	0.028	0.193	0.614	—

Net metabolic rates are all expressed in units of W kg ^−1^ (mean ± s.e.m.). *P* < 0.05 represents statistical significance

There was a large change in metabolic power during powered minutes 3-6 across sessions. During session 1, subjects had a net metabolic power of 3.72±0.18 W kg ^−1^, a reduction of 5.6 % compared to the 1^st^ unpowered condition. By session 3, net metabolic power was 3.14±0.11 W kg ^−1^, a reduction of 14.2 % compared to the 1^st^ unpowered condition. Statistically, there was a significant reduction in net metabolic power during powered minutes 3-6 across the three sessions (*P*=0.028).

### Dynamically adjusted gain

By the end of session 3, our adaptive controller chose gains that resulted in *β*=1.50±0.14 (mean ± s.e.m. between subjects; we averaged *β*_*i*_ over the final three minutes of the powered session to calculate *β*). The average gain values from the final three minutes of each session showed no significant difference across sessions (*P*=0.273).

### Electromyography

**Fig. 4:**
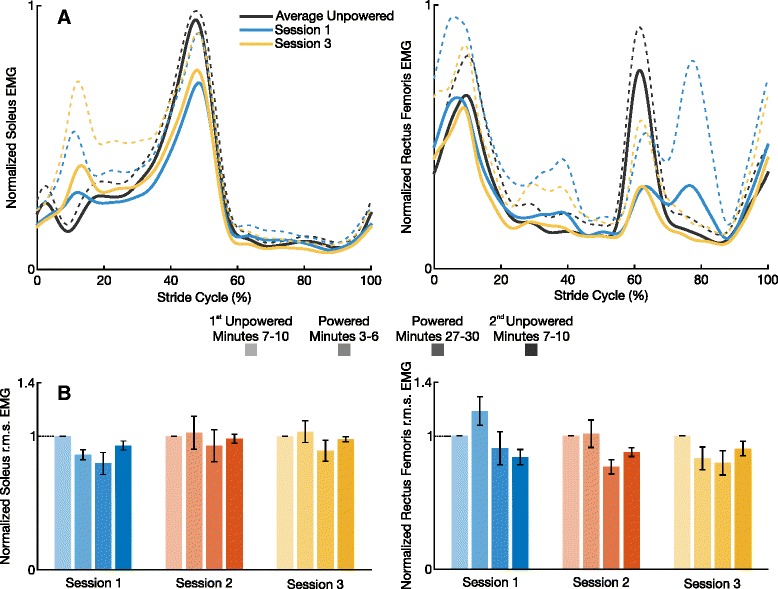
Soleus and Rectus Femoris EMG. **a** The mean soleus and mean rectus femoris EMG linear envelope (high-pass cutoff frequency of 35 Hz and low-pass cutoff frequency of 10 Hz) of eight subjects is represented by the solid lines and + 1 s.d. is represented by the dashed lines. **b** The mean soleus and mean rectus femoris r.m.s. of rectified EMG for four time intervals is indicated by the colored bars across all three sessions. Error bars represent ±1 s.e.m. Each subject’s r.m.s. values were normalized to the corresponding session’s 1^st^ unpowered r.m.s. value prior to averaging

During session 1, subjects quickly reduced their soleus activation levels (Fig. 4 and Additional file 2: Table A2). At the beginning of the powered condition of session 1, subjects reduced their soleus r.m.s. EMG by 13.8±3.8 *%* compared to the end of the 1^st^ unpowered condition. By the end of that same session, subjects had achieved a soleus r.m.s. EMG reduction of 20.3±8.2 *%* (28.0±6.8 *%* reduction in peak linear envelope). Contrary to previous studies, subjects preferred to increase their soleus recruitment with additional training sessions. By the end of session 3, subjects were walking with a soleus r.m.s. EMG reduction of only 10.8±7.9 *%* (21.5±4.8 *%* reduction in peak linear envelope). The medial gastrocnemius EMG showed no significant change during testing.

Across testing sessions, subjects adapted to use less rectus femoris recruitment when walking in the powered exoskeleton (Fig. 4 and Additional file 2: Table A2). By the end of the powered condition of session 3, subjects had adapted to reduce their rectus femoris r.m.s. EMG by 20.2±9.2 *%* compared to the 1^st^ unpowered condition. As subjects learned to walk in the exoskeleton, their rectus femoris activity decreased across sessions during the powered minutes 3–6 (*P*=0.005). The most noticeable change was the reduction in peak EMG activity shown by Fig. 4. By session 3, subjects were able to reduce their peak rectus femoris activation level around toe off by 43.8±13.8 *%* compared to the 1^st^ unpowered condition. Unlike the rectus femoris EMG, the vastus lateralis EMG showed no r.m.s. reduction during powered walking. The biceps femoris long head EMG r.m.s. values showed significant reductions during each session (all *P* <0.05), yet the reduction observed during the end of the powered condition lasted through the end of the 2^nd^ unpowered condition (Additional file 2: Table A2).

### Joint kinematics

**Fig. 5:**
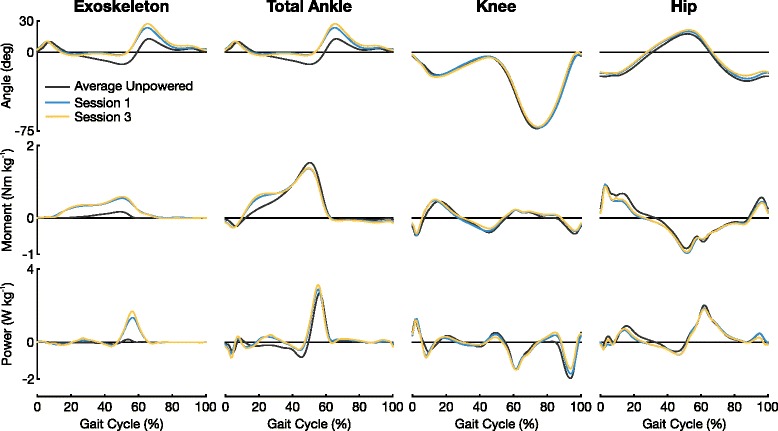
Joint Kinematics, Dynamics, and Power. Mean joint angles, moments, and powers from eight subjects. Joint dynamics and power have been normalized by subject mass. In the kinematics and dynamics plots all positive numbers represent extension while all negative numbers represent flexion

Subjects had the largest change in joint kinematics at the ankle when comparing powered to unpowered conditions (Fig. 5). A linear regression between ankle kinematics from the end of the powered condition in session 3 and the 1^st^ unpowered condition of that same session had an *R*^2^ value of 0.58±0.11. This lack of correlation between the two conditions is due to the fact that subjects plantar flexed ∼8–9° more throughout the mid and late stance phase (30–60 % gait cycle). Subjects continued to increase peak plantar flexion from session 1 (23.5°) to session 3 (27.3°). The powered peak plantar flexion values are large compared to the 1^st^ unpowered condition (12.9°).

Little change was observed in the knee and hip kinematics. All hip and knee linear regressions comparing the end of the powered condition to the 1^st^ unpowered condition of each session had *R*^2^ values greater than 0.97.

### Joint mechanics

The relationship between subjects’ actuation control signal magnitude and exoskeleton torque output was approximately linear with an *R*^2^ value of 0.74±0.13 by the end of session 3. The mean total moment at the ankle (biological and exoskeleton) increased ∼0.16–0.18 Nm kg^-1^ (∼47.8 %) during the early to mid stance phase (0–30 % gait cycle) when comparing the end powered conditions to the average unpowered condition (Fig. 5). This increase in total ankle plantarflexion moment during the early to mid stance phase corresponds with a decrease in hip flexion muscle moment. Subjects experienced a decrease in mean hip flexion muscle moment ∼0.14–0.15 Nm kg^-1^ (∼31.7 %) during this phase of the gait. There was little change in knee joint dynamics.

**Fig. 6:**
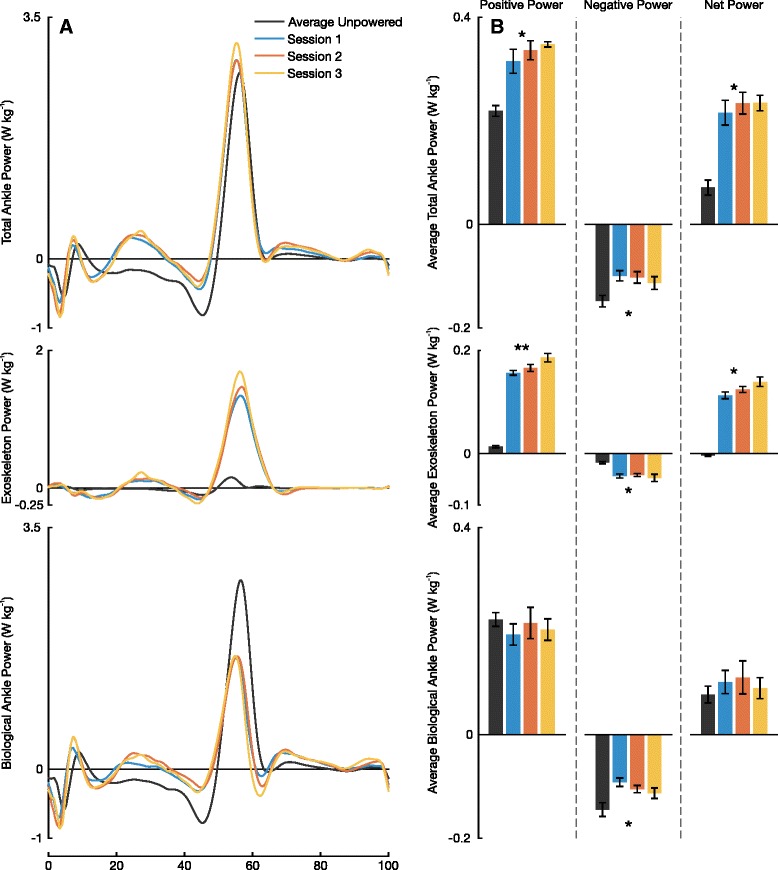
Breakdown of Ankle Power. **a** Mean total ankle power, exoskeleton power, and biological ankle power from eight subjects across all three sessions. The exoskeleton power was calculated from ankle kinematics and force outputs. The biological power was calculated by subtracting the exoskeleton power from the total ankle power. **b** Average power plots of positive, negative, and net power for total ankle power, exoskeleton power, and biological ankle power. All error bars represent ±1 s.e.m. An astrix represent significance across all four conditions (ANOVA, *P* <0.05) and a double astrix represents significance in both all four conditions as well as just across sessions 1–3 (ANOVA, *P* <0.05)

**Fig. 7:**
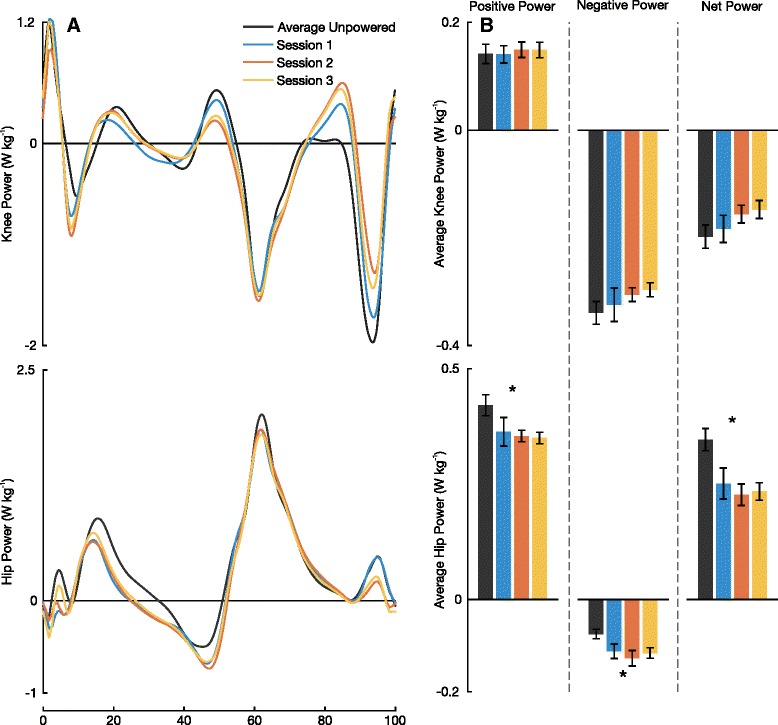
Breakdown of Knee and Hip Power. **a** Mean knee power and mean hip power from eight subjects. **b** Average power plots of positive, negative, and net power for knee and hip power. An astrix represent significance across all four conditions (ANOVA, *P* <0.05)

Subjects increased positive average total ankle power when the exoskeleton was powered (*P*=0.001; Fig. 6). Most noticeably, subjects walked with a 0.13±0.01 W kg^-1^ (65.8±8.9 %) increase in positive average total ankle power by session 3 relative to the average unpowered condition. Across training sessions, subjects increased their positive ankle exoskeleton power as they adapted to the device (*P*=0.019). Subjects had no significant change in net biological power output between powered and average unpowered conditions (*P*=0.614). There was no significant difference in average net knee power between powered and average unpowered conditions (*P*=0.195), yet a decreasing trend of the magnitude was observed. Between the average unpowered condition and the end of session 3’s powered condition, there was a 25.4 *%* reduction in the magnitude of the average net knee power. There were significant differences in average positive hip power between powered and average unpowered conditions (*P*=0.003). By session 3, subjects walked with an average positive hip power 0.06±0.01 W kg^-1^ (14.7±2.5 %) less than that of the 1^st^ average unpowered condition (Fig. 7).

**Fig. 8:**
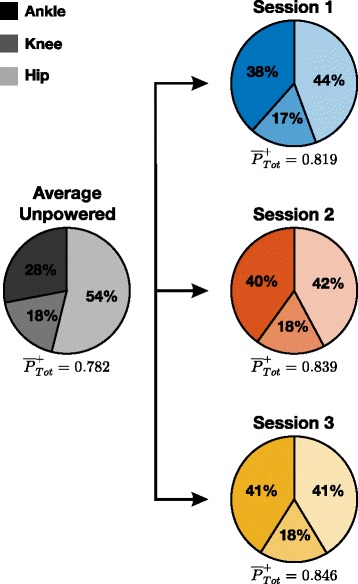
Total Positive Power Contributions. Mean ankle (dark), knee (medium), and hip (light) percent contributions to total positive power from eight subjects during unpowered and powered walking

Subjects increased the amount of average total positive power, $\overline {P}_{\textit {Tot}}^{+}$, from the average unpowered condition to the end of the powered sessions (*P*=0.009). Additionally subjects altered percent contributions of the ankle and hip joint to $\overline {P}_{\textit {Tot}}^{+}$ (*P*=0.002 and *P*=0.002 respectively; Fig. 8). There was no significant change in percent contributions from the knee between conditions (*P*=0.165). Percent contributions from the ankle increased from 27.7±1.9 *%* to 41.2±1.0 *%* between the average unpowered condition and the end of the powered condition of session 3. Percent contributions from the hip decreased from 52.8±1.6 *%* to 41.3±0.9 *%* between the average unpowered condition and the end of the powered condition of session 3.

## Discussion

The results from this study support our hypothesis that subjects would learn to reduce their energetic cost when walking in the robotic ankle exoskeletons. By the end of the session 3, subjects required 3.01±0.08 W kg ^−1^ to walk in the powered device. This result is comparable to that of previous studies using a traditional proportional myoelectric controller [15]. An important difference between our metabolic results and that of previous studies is that all eight of our subjects experienced a metabolic reduction by the end of session 1’s powered condition. In previous studies, the mean metabolic reduction by the end of session 1’s powered condition was approximately zero. Additionally, in previous work subjects had to complete three full training sessions before additional training had no effect metabolic power reduction. In our current study there was no statistically significant difference in net metabolic power reduction at the end of the powered condition across sessions (*P*=0.193). Although the mean metabolic values at the end of each session’s powered condition suggests slight training effects, the percent reduction between the first unpowered condition and end of the powered condition on session 1 was 16.2 *%* were on session 3 it was 17.8 *%*. These results suggests that the learning rate with an adaptive proportional myoelectric controller is faster than that of a traditional proportional myoelectric controller.

Despite no significant difference in net metabolic power reduction at the end of the powered condition across sessions, the rate at which subjects reached this net metabolic power reduction increased with additional training sessions. This is made evident by the significant differences in net metabolic power during the beginning of the powered condition across sessions (*P*=0.028; Fig. 3 and Table 2). These metabolic results show that an adaptive gain proportional myoelectric controller can positively assist users. It is important to note that the control scheme presented here is not the first variation on the traditional proportional myoelectric control algorithm [36]; however, to the best of our knowledge it is the first to implement an adaptive gain.

In addition to the metabolic reductions, our results also suggest that subjects preferred a *β* value *smaller* than that used in our previous work (*β*=1.50±0.14 versus a constant *β*=2). We found that subjects had no statistical difference in final *β* gains between sessions (*P*=0.273) which suggests the gain converged to a steady state value after only one session. According to Eq. 7, this smaller gain should lead to a *larger* total joint activity compared to unassisted walking ($\mathbf {X}_{\textit {Tot}} > \mathbf {\overline {X}}_{\textit {Bio}}$). This relationship might seem unintuitive at first, but it is important to note that in both cases the exoskeleton is operated close to its maximum capacity of $\mathbf {\widehat {X}}_{\textit {Exo}}$. Given a smaller gain *β*, the user’s contribution **X**_*Bio*_ was larger than that of previous studies without oversaturating **X**_*Exo*_. It is this contribution from the user that leads to a larger total joint activity at the ankle. Our prediction of increased **X**_*Tot*_ as a result of a smaller *β* manifested itself in this study by an increase in positive average total ankle power. Positive average total ankle power increased from 0.21 to 0.35 W kg^-1^ between unpowered and powered conditions on session 3. The exoskeleton provided 0.17 W kg^-1^ additional average positive power, while the biological average positive power was reduced by only 0.02 W kg^-1^. We did not observe an increase in total ankle power with our previous work using a static gain proportional myoelectric controller. Our methodology for tuning *β* in the past may have constrained users to using levels of total ankle power no larger than that of unassisted walking in the device.

This increase in positive average total ankle power led to significant changes in hip joint mechanics. Positive average hip power decreased from 0.41 to 0.35 W kg^-1^ between unpowered and powered conditions on session 3. Additionally, our results show that subjects chose to increase ankle contribution to total positive power (27.7 to 41.2 *%*) in exchange for a decrease in hip contribution (52.8 to 41.3 *%*) between these conditions. We acknowledge that the baseline of the unpowered condition is shifted from walking without an exoskeleton most likely due to the added mass of the device. As a point of reference, Farris et al. showed that in healthy subjects walking without any exoskeleton at 1.25 ms^-1^ (compared to 1.2 ms^-1^ in this study) about 46 *%* of the total average positive power comes from the ankle while 40 *%* comes from the hip [34]. Although the percent contributions of power at the end of session 3 in this study look similar to those reported by Farris et al., we would not conclude that subjects adapted back to normal unassisted gait dynamics. We would not make this conclusion due to the large differences in power and moment profiles of each individual joint from this study compared to that of previously reported profiles of healthy unassisted walking [34, 37]. Our results emphasize that replicating unassisted joint mechanics with assistive devices may not be the best approach to lowering metabolic power. We also observed a trade-off in soleus EMG activity and rectus femoris EMG activity. This result agrees with previous studies such that ankle assistance can lead to decreases in activity at muscles not associated with the ankle [38, 39].

Research has shown that a trade-off between ankle and hip mechanics exists in unassisted locomotion. The possibility of redistributing joint powers has been shown for example by Lewis et al. [40]. When subjects were asked to walk with an increased ankle push off, the power at their hip decreased. However, little has been said about the energetic implications of this trade-off with human subject testing. In 2002, Art Kuo showed in simulation that increasing work at the ankle can be energetically economical in comparison to doing so at the hip [41]. He further hypothesized that it is only biological limitations that prevent us from using more ankle work in practice. Our results might point in the same direction. During unpowered walking, the ratio of hip to ankle contribution that we observe is larger than that reported in previous literature [34, 42]. This may be a consequence of the increase in required total positive joint power that results from the mass of the exoskeletons which is added distally to the legs. We believe that this additional power is primarily produced at the hip because there exists a biological limitation preventing the ankle from comfortably providing more positive power. With the added power of the exoskeleton, however, subjects were able to increase contributions from the ankle and reduce the effort put forth at the hip. Our findings that an ankle exoskeleton can reduce effort at the hip can potentially be applied to musculoskeletal hip rehabilitation. Given that subjects showed large reductions in average positive hip power, an ankle exoskeleton could be a viable option for those in need of hip assistance yet more testing is necessary to say for certain.

## Conclusion

This study used an adaptive proportional myoelectric controller on bilateral ankle exoskeletons to test if users could adapt to the controller to reduce metabolic power and see what *β* gain they chose when given an adaptive controller. Subjects demonstrated that a significant metabolic reduction can be met after only one day of training. Subjects adapted to a *β* gain smaller than that used in previous work with traditional proportional myoelectric controllers. This smaller *β* gain allowed subjects increased amounts of total ankle power compared to unassisted walking and resulted in reduced power output at the hip.

More research is needed to be done in adaptive control of assistive devices to gain a better understanding of how subjects co-adapt with these systems. However, we believe that an adaptive nature of control parameters will be key to developing better assistive devices.

## Additional files

Additional file 1
**Figure A1.** Here we have a cartoon example of how the controller reacts given a subject’s adaptation in the device. **(A)** The controller is turned off during unpowered walking and the user receives no actuation. **(B)** When the controller is turned on, the finite impulse response filter begins to initialize with strides causing the mapping gain, *G*_*i*_, to increase. This increase in *G*_*i*_ causes an increase in the exoskeleton activity, **X**_*Exo*_. **(C)** The user then begins to adapt to the actuation by decreasing their biological activity, **X**_*Bio*_. During this time the adaptive controller compensates for the decreased biological activity by increasing *β*_*i*_ and thus the mapping gain *G*_*i*_. This increase in *G*_*i*_ brings the exoskeleton activity back toward the saturation limit $\mathbf {\widehat {{X}}}_{\textit {Exo}}$. **(D)** The user then holds their adapted biological activity at some steady state value. *β*_*i*_ and *G*_*i*_ settle at their steady state values *β*_*ss*_ and *G*_*ss*_, respectively. (PDF 177kb)

Additional file 2
**Table A2.** Root mean square electromyography data for all recorded muscles. (PDF 56kb)

## References

[1] Dollar AM, Herr H (2008). Lower extremity exoskeletons and active orthoses: challenges and state-of-the-art. IEEE Trans Robot.

[2] Jimenez-Fabian R, Verlinden O (2012). Review of control algorithms for robotic ankle systems in lower-limb orthoses, prostheses, and exoskeletons. Med Eng Phys.

[3] Blaya JA, Herr H (2004). Adaptive control of a variable-impedance ankle-foot orthosis to assist drop-foot gait. IEEE Trans Neural Syst Rehabil Eng.

[4] Kazerooni H, Racine J, Huang L, Steger R. On the control of the berkeley lower extremity exoskeleton (BLEEX). In: Robotics and automation, 2005. ICRA 2005. Proceedings of the 2005 IEEE international conference on. IEEE: 2005.

[5] Hollander KW, Ilg R, Sugar TG, Herring D (2006). An efficient robotic tendon for gait assistance. J Biomech Eng.

[6] Banala SK, Kim SH, Agrawal SK, Scholz JP (2009). Robot assisted gait training with active leg exoskeleton (alex). IEEE Trans Neural Syst Rehabil Eng.

[7] Li D, Becker A, Shorter KA, Bretl T, Hsiao-Wecksler E (2011). Estimating system state during human walking with a powered ankle-foot orthosis. IEEE/ASME Trans Mechatron.

[8] Malcolm P, Derave W, Galle S, De Clercq D (2013). A simple exoskeleton that assists plantarflexion can reduce the metabolic cost of human walking. PloS One.

[9] Mooney LM, Rouse EJ, Herr HM (2014). Autonomous exoskeleton reduces metabolic cost of human walking during load carriage. J Neuroeng Rehabil.

[10] Ferris DP, Sawicki GS, Daley MA (2007). A physiologist’s perspective on robotic exoskeletons for human locomotion. Int J Humanoid Robot.

[11] Hogan N (1976). A review of the methods of processing emg for use as a proportional control signal. Biomed Eng.

[12] Ferris DP, Lewis CL. Robotic lower limb exoskeletons using proportional myoelectric control. In: Engineering in Medicine and Biology Society, 2009. EMBC 2009. Annual International Conference of the IEEE. IEEE: 2009. 10.1109/IEMBS.2009.5333984 PMC2833282 19964579

[13] Cavanagh P, Komi P (1979). Electromechanical delay in human skeletal muscle under concentric and eccentric contractions. Eur J Appl Physiol Occup Physiol.

[14] Gordon KE, Ferris DP (2007). Learning to walk with a robotic ankle exoskeleton. J Biomech.

[15] Sawicki GS, Ferris DP (2008). Mechanics and energetics of level walking with powered ankle exoskeletons. J Exp Biol.

[16] Kao PC, Ferris DP (2009). Motor adaptation during dorsiflexion-assisted walking with a powered orthosis. Gait Posture.

[17] Kinnaird CR, Ferris DP (2009). Medial gastrocnemius myoelectric control of a robotic ankle exoskeleton. IEEE Trans Neural Syst Rehabil Eng.

[18] Kao PC, Lewis CL, Ferris DP (2010). Invariant ankle moment patterns when walking with and without a robotic ankle exoskeleton. J Biomech.

[19] Gordon KE, Kinnaird CR, Ferris DP (2013). Locomotor adaptation to a soleus emg-controlled antagonistic exoskeleton. J Neurophys.

[20] Alexander RM (1996). Optima for animals.

[21] Sparrow WA (2000). Energetics of human activity.

[22] Donelan JM, Kram R (2001). Mechanical and metabolic determinants of the preferred step width in human walking. Proc R Soc Lond Ser B Biol Sci.

[23] Umberger BR, Martin PE (2007). Mechanical power and efficiency of level walking with different stride rates. J Exp Biol.

[24] Finley JM, Bastian AJ, Gottschall JS (2013). Learning to be economical: the energy cost of walking tracks motor adaptation. J Physiol.

[25] Ferris DP, Czerniecki JM, Hannaford B (2005). An ankle-foot orthosis powered by artificial pneumatic muscles. J Appl Biomech.

[26] Ferris DP, Gordon KE, Sawicki GS, Peethambaran A (2006). An improved powered ankle–foot orthosis using proportional myoelectric control. Gait Posture.

[27] Shadmehr R, Holcomb HH (1997). Neural correlates of motor memory consolidation. Science.

[28] Brockway J (1987). Derivation of formulae used to calculate energy expenditure in man. Hum Nutr Clin Nutr.

[29] Griffin TM, Roberts TJ, Kram R (2003). Metabolic cost of generating muscular force in human walking: insights from load-carrying and speed experiments. J Appl Physiol.

[30] Brooks GA, Fahey TD, White TP (1996). Exercise physiology: Human bioenergetics and its applications.

[31] Winter D, Yack H (1987). Emg profiles during normal human walking: stride-to-stride and inter-subject variability. Electroencephalogr Clin Neurophysiol.

[32] Delp SL, Anderson FC, Arnold AS, Loan P, Habib A, John CT (2007). Opensim: open-source software to create and analyze dynamic simulations of movement. IEEE Trans Biomed Eng.

[33] Hicks J, Seth A, Hamner S, Demers M. Simulation with OpenSim - Best Practices. http://simtk-confluence.stanford.edu:8080/display/OpenSim/Simulation+with+OpenSim+-+Best+Practices. Accessed 1 April 2015.

[34] Farris DJ, Sawicki GS (2011). The mechanics and energetics of human walking and running: a joint level perspective. J R Soc Interface.

[35] Collins SH, Wiggin MB, Sawicki GS (2015). Reducing the energy cost of human walking using an unpowered exoskeleton. Nature.

[36] Takahashi KZ, Lewek MD, Sawicki GS (2015). A neuromechanics-based powered ankle exoskeleton to assist walking post-stroke: a feasibility study. J Neuroeng Rehabil.

[37] Winter DA (1984). Kinematic and kinetic patterns in human gait: variability and compensating effects. Hum Mov Sci.

[38] Galle S, Malcolm P, Derave W, De Clercq D (2015). Uphill walking with a simple exoskeleton: Plantarflexion assistance leads to proximal adaptations. Gait Posture.

[39] Galle S, Malcolm P, Collins SH, Speeckaert J, De Clercq D. Optimizing robotic exoskeletons actuation based on human neuromechanics experiments: Interaction of push-off timing and work. Cambridge, MA, USA: 7th Int. Symposium on Adaptive Motion of Animals and Machines (AMAM): 2015.

[40] Lewis CL, Ferris DP (2008). Walking with increased ankle pushoff decreases hip muscle moments. J.

[41] Kuo AD (2002). Energetics of actively powered locomotion using the simplest walking model. J Biomech Eng.

[42] Sawicki GS, Lewis CL, Ferris DP (2009). It pays to have a spring in your step. Exerc Sport Sci Rev.

