# Supplementary figures and images for: Learning to walk with an adaptive gain proportional myoelectric controller for a robotic ankle exoskeleton
Source: J Neuroeng Rehabil. 2015 Nov 4;12:97. doi: 10.1186/s12984-015-0086-5 (PMC4634144; doi:10.1186/s12984-015-0086-5)

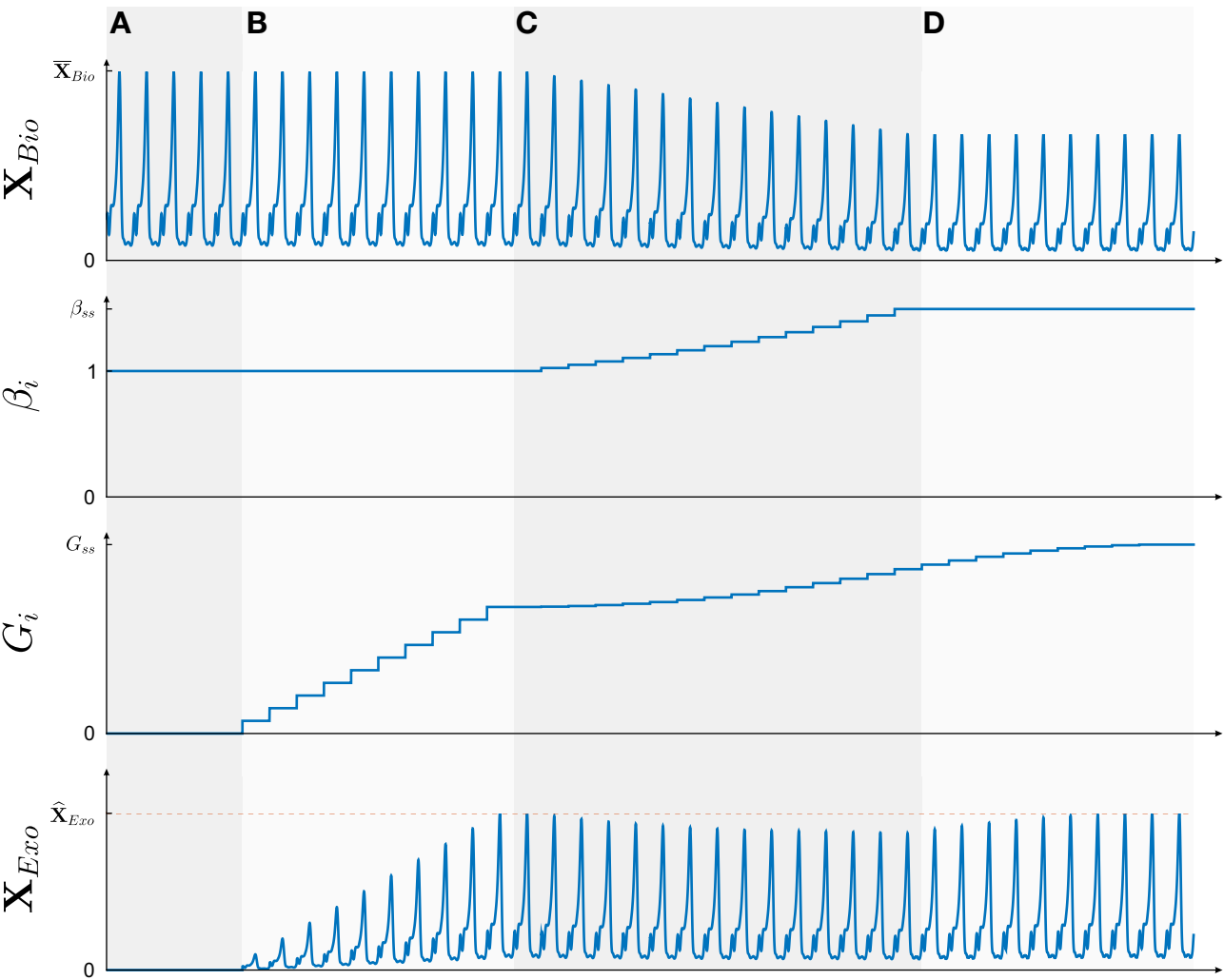

Supplement: Additional file 1 — Figure A1. Here we have a cartoon example of how the controller reacts given a subject’s adaptation in the device. (A) The controller is turned off during unpowered walking and the user receives no actuation. (B) When the controller is turned on, the finite impulse response filter begins to initialize with strides causing the mapping gain, G i, to increase. This increase in G i causes an increase in the exoskeleton activity, X Exo. (C) The user then begins to adapt to the actuation by decreasing their biological activity, X Bio. During this time the adaptive controller compensates for the decreased biological activity by increasing β i and thus the mapping gain G i. This increase in G i brings the exoskeleton activity back toward the saturation limit $\mathbf {\widehat {{X}}}_{\textit {Exo}}$X^Exo. (D) The user then holds their adapted biological activity at some steady state value. β i and G i settle at their steady state values β ss and G ss, respectively. (PDF 177kb) [file 12984_2015_86_MOESM1_ESM.pdf]
