# Supplementary material for: Learning to walk with an adaptive gain proportional myoelectric controller for a robotic ankle exoskeleton
Source: J Neuroeng Rehabil. 2015 Nov 4;12:97. doi: 10.1186/s12984-015-0086-5 (PMC4634144; doi:10.1186/s12984-015-0086-5)
Supplement: Additional file 2 — Table A2. Root mean square electromyography data for all recorded muscles. (PDF 56kb) [file 12984_2015_86_MOESM2_ESM.pdf]

Table A2 Root mean square electromyography for muscles of interest

	1st Unpowered Minutes 7-10	Powered Minutes 3-6	Powered Minutes 27-30	2nd Unpowered Minutes 7-10	Within Session *P*-Value
Soleus r.m.s.					
Session 1	1.00 ± 0.00	0.86 ± 0.04	0.80 ± 0.08	0.93 ± 0.03	0.073
Session 2	1.00 ± 0.00	1.03 ± 0.12	0.93 ± 0.12	0.98 ± 0.03	0.307
Session 3	1.00 ± 0.00	1.04 ± 0.08	0.89 ± 0.08	0.98 ± 0.02	0.119
Across Session *P*-Value	1.000	0.251	0.478	0.432	
Tibialis Anterior r.m.s.					
Session 1	1.00 ± 0.00	1.20 ± 0.03	1.07 ± 0.05	1.01 ± 0.04	0.005
Session 2	1.00 ± 0.00	1.10 ± 0.05	0.92 ± 0.03	0.99 ± 0.04	0.032
Session 3	1.00 ± 0.00	1.04 ± 0.05	0.89 ± 0.06	1.00 ± 0.04	0.354
Across Session *P*-Value	1.000	0.130	0.118	0.947	
Medial Gastrocnemius r.m.s.					
Session 1	1.00 ± 0.00	1.05 ± 0.07	0.98 ± 0.09	0.99 ± 0.05	0.317
Session 2	1.00 ± 0.00	1.02 ± 0.11	0.96 ± 0.12	0.98 ± 0.03	0.783
Session 3	1.00 ± 0.00	1.03 ± 0.09	0.94 ± 0.07	0.98 ± 0.02	0.252
Across Session *P*-Value	1.000	0.885	0.885	0.885	
Biceps Femoris Long Head r.m.s.					
Session 1	1.00 ± 0.00	1.12 ± 0.10	0.82 ± 0.05	0.82 ± 0.05	0.002
Session 2	1.00 ± 0.00	1.02 ± 0.10	0.76 ± 0.08	0.80 ± 0.06	0.039
Session 3	1.00 ± 0.00	1.19 ± 0.14	0.83 ± 0.09	0.87 ± 0.04	0.014
Across Session *P*-Value	1.000	0.115	0.008	0.672	
Vastus Lateralis r.m.s.					
Session 1	1.00 ± 0.00	1.18 ± 0.06	1.02 ± 0.08	0.93 ± 0.02	0.002
Session 2	1.00 ± 0.00	1.15 ± 0.06	1.04 ± 0.11	0.95 ± 0.04	0.178
Session 3	1.00 ± 0.00	1.13 ± 0.06	1.01 ± 0.07	0.99 ± 0.04	0.127
Across Session *P*-Value	1.000	0.670	0.912	0.427	
Rectus Femoris r.m.s.					
Session 1	1.00 ± 0.00	1.19 ± 0.11	0.91 ± 0.12	0.84 ± 0.06	0.008
Session 2	1.00 ± 0.00	1.02 ± 0.10	0.77 ± 0.05	0.88 ± 0.03	0.058
Session 3	1.00 ± 0.00	0.83 ± 0.09	0.80 ± 0.09	0.90 ± 0.05	0.350
Across Session *P*-Value	1.000	0.005	0.555	0.373	

Values (means ± s.e.m.) are root mean square (r.m.s.) average from the whole walking stride normalized to the first unpowered condition of the corresponding session. $P<0.05$ represents statistical significance.
